# Immune Modulation in Xenotransplantation

**DOI:** 10.1007/s00005-014-0317-7

**Published:** 2014-10-30

**Authors:** Magdalena Boksa, Joanna Zeyland, Ryszard Słomski, Daniel Lipiński

**Affiliations:** 1 Department of Biochemistry and Biotechnology, Poznań University of Life Sciences, Dojazd 11, 60-632 Poznań, Poland; 2 Institute of Human Genetics, Polish Academy of Sciences, Poznań, Poland

**Keywords:** Xenotransplantation, Transgenic pig, Hyperacute rejection, Cytotoxicity, Coagulation

## Abstract

The use of animals as donors of tissues and organs for xenotransplantations may help in meeting the increasing demand for organs for human transplantations. Clinical studies indicate that the domestic pig best satisfies the criteria of organ suitability for xenotransplantation. However, the considerable phylogenetic distance between humans and the pig causes tremendous immunological problems after transplantation, thus genetic modifications need to be introduced to the porcine genome, with the aim of reducing xenotransplant immunogenicity. Advances in genetic engineering have facilitated the incorporation of human genes regulating the complement into the porcine genome, knockout of the gene encoding the formation of the Gal antigen (α1,3-galactosyltransferase) or modification of surface proteins in donor cells. The next step is two-fold. Firstly, to inhibit processes of cell-mediated xenograft rejection, involving natural killer cells and macrophages. Secondly, to inhibit rejection caused by the incompatibility of proteins participating in the regulation of the coagulation system, which leads to a disruption of the equilibrium in pro- and anti-coagulant activity. Only a simultaneous incorporation of several gene constructs will make it possible to produce multitransgenic animals whose organs, when transplanted to human recipients, would be resistant to hyperacute and delayed xenograft rejection.

## Introduction

At present, transplantation of cells, tissues and organs is an effective and frequently the only method to save the lives of patients with end-stage organ failure, victims of accidents and patients suffering from cancers of the hematopoietic systems or complex immunodeficiencies. The main problem in contemporary transplantology is connected with the existing disproportion between the volume of available organs and the number of patients qualified for transplantations (Mulligan et al. 2008). Neither rationalization of the systems for registering and distributing organs for transplantation, nor improving the health in society by promoting healthy lifestyles are sufficiently effective to alleviate the critical deficit of available organs and tissues for transplantations. This situation has persisted for many years. The problem may be solved, thanks to the use of animal-origin cells, tissues and organs for transplantation, i.e. xenotransplants. The domestic pig (*Sus scrofa*) is an optimal donor for transplant organs due to its anatomical and physiological similarities to humans, unlimited availability, good breeding potential, short period to reproductive maturity, relatively short pregnancy and high number of offspring (Cooper et al. 2002). However, the considerable phylogenetic distance between this potential donor and the human recipients causes tremendous immunological problems following transplantation. It is therefore necessary to introduce genetic modifications and incorporate human genes into the porcine genome, which may reduce immunogenicity of xenotransplantations.

The primary cause of transplant rejection in the pig-human system is connected with the Gal antigen (Galα1,3Gal) present in glycolipids and glycoproteins found on the surface of porcine cells. The Gal antigen is formed as a result of the galactose molecule attaching to *N*-acetyllactosamine (*N*-lac) with the α1,3-glycoside bond and involving the α1,3-galactosyltransferase enzyme (Sandrin and McKenzie 1994). Neither the enzyme nor the sugar unit is found in humans or the Old World monkeys (Galili et al. 1988). Identification and binding of the Gal antigen by xenoreactive antibodies triggers a series of reactions, which lead to transplant rejection as a result of “hyperacute rejection” (Daniels and Platt 1997).

## Hyperacute Rejection

The necessary condition to provide organs suitable for interspecific transplantations is to remove the Gal antigen from the surface of xenotransplant cells. In 2001 two cooperating research teams, one headed by Randall Prather and the other composed of researchers employed at Immerge BioTherapheutics, managed to produce pigs with the GGTA1 gene inactivated in the heterozygous system (Dai et al. 2002). A similar achievement was recorded in the same year by PPL Therapeutics (Lai et al. 2002). In July 2002 the first piglets with two knockout alleles of the GGTA1 gene were born at PPL Therapeutics (Phelps et al. 2003), while a similar success was reported by researchers from Immerge BioTherapeutics. A small, but detectable amount of the Gal antigen is found on the surface of fibroblast cells from porcine embryos with the inactivated gene encoding α1,3-galactosyltransferase in the homozygous system (Sharma et al. 2003). It is estimated that cells synthesize the Gal antigen at 1–2 %, in comparison to the amount of the Gal antigen produced in heterozygous cells in relation to the inactivated gene. In 2005 the first xenotransplantation was performed with the use of porcine organs with the inactivated α1,3-galactosyltransferase gene. Pig hearts were transplanted to baboons subjected to immunosuppression. The mean length of xenotransplant survival was 92 days, with one of the hearts functioning for 179 days (Kuwaki et al. 2005; Tseng et al. 2005a; Yamada et al. 2005). None of the organs was rejected as a result of a hyperacute immune reaction. Transplant rejections were caused by other, less intense reactions.

An alternative to the strategy aimed at rendering the GGTA1 gene inactivate is to incorporate in the porcine genome some human genes encoding enzymes which modify the oligosaccharide structure of glycoproteins and glycolipids found on the cell surface in graft donors. The activity of such enzymes should result in the Gal antigen being eliminated or markedly reduced. The enzyme α1,2-fucosyltransferase (H transferase) uses the same acceptor, *N*-lac as α1,3-galactosyltransferase (Larsen et al. 1990). This enzyme catalyzes the addition of fucose units, which leads to the formation of a “H structure”. Additional copies of the human H transferase gene incorporated in the donor organisms facilitate modification of surface proteins in donor cells, which limits the immunogenicity of organs for transplantation (Costa et al. 1999; Sharma et al. 1996). Both strategies aim to inhibit hyperacute xenograft rejection and were successfully combined. The result was transgenic pigs with the inactivated GGTA1 gene and expressing human H transferase (Ramsoondar et al. 2003). Apart from preventing hyperacute rejection, human transferase expressed in porcine cells might also prevent or weaken delayed xenograft rejection. The sensitivity of porcine endothelial cells to lysis caused by the involvement of human natural killer (NK) cells is reduced as a result of human H transferase expression (Artrip et al. 1999). Expression of human H transferase in the endothelial cells of pigs also limits adhesion and activation of monocytes (Kwiatkowski et al. 1999). In this way the strategy involving human H transferase causes reduced binding of anti-Gal specific antibodies, thus preventing the development of hyperacute xenograft rejection. This strategy also reduces the response involving NK cells and monocytes. It is assumed that in the case of certain types of cells, expression of human H transferase may prove sufficient to provide protection against rejection, whether a hyperacute or delayed reaction (Costa et al. 1999).

Another mechanism facilitating a reduction in the amount of Gal antigen on the surface of transplant donor cells uses α-galactosidase (GLA), which catalyzes the removal of the α-d-galactose unit from the Gal antigen found on the cell surface. Incorporating the gene encoding the human GLA enzyme into the porcine genome made it possible to produce organs for transplantations with a reduced amount of the Gal epitope on the cell surface and a limited complement-dependent cytotoxicity in comparison with the control cells (Zeyland et al. 2013). Since human GLA does not eliminate all α-d-galactose units from the Gal antigen, the suggestion is to simultaneously use GLA and α1,2-fucosyltransferase. The additive effect of applying both enzymes was observed in the case of co-transfection of different types of cells with genes encoding human GLA and α1,2-fucosyltransferase. In such a situation the expression of the Gal antigen is not observed on the cell surface (Jia et al. 2004; Osman et al. 1997; Yan et al. 2003). However, in double transgenic animals with confirmed integration of both transgenes in the heterozygous system, a complete elimination of the Gal antigen from the cell surface was not observed (Zeyland et al. 2014).

The incompatibility of porcine complement regulatory proteins (CRPs) on donor endothelialium with a human complement resulted in uncontrolled complement activation. Therefore, the presence of human CRPs on the pig endothelium could stop complement activation in the recipient. The complement is mainly regulated by CD46 (membrane cofactor protein), CD59 (membrane inhibitor of reactive lysis) and CD55 (decay accelerating factor) factors. These proteins belong to a family of structurally and functionally similar proteins which block complement activation and prevent the formation of a complex attacking the cell membranes. To date, pigs that express human CD46 (hCD46), CD55, CD59 have been produced (Chen et al. 1999; Cozzi et al. 2000; Deppenmeier et al. 2006; Diamond et al. 1996; Fodor et al. 1994; Langford et al. 1994; Niemann et al. 2001; Waterworth et al. 1998; Zhou et al. 2005). In 2005, a team of researchers headed by McGregor managed to obtain the longest to date mean survival time of a xenograft originating from monotransgenic animals in the pig-to-primate system (McGregor et al. 2005). Hearts coming from genetically modified pigs with additional copies of the gene encoding the hCD46 factor were transplanted to baboons and survived on average 96 days. The maximum transplant survival time was 137 days. Transplant survival time was influenced not only by the type of genetic modification applied, but also the type and doses of drugs administered after the procedure. It transpired that an increased dose of immunosuppressants (tacrolimus, sirolimus) and a reduced dose of anticoagulants considerably extended the mean survival time of a xenogenic transplant in comparison to that of transplants in groups of animals subjected to other types of therapy (Byrne et al. 2006). Organs coming from such modified animals are protected against attack from the human complement and hyperacute rejection, but are still subjected to processes of delayed xenograft rejection. To a considerable degree, prevention against the further development of xenograft rejection processes is dependent on the administration of immunosuppressants. The newest preparations should be selective in their action so that only processes connected with xenograft rejection could be blocked and which would ensure complete immune response against developing infections (Ingelfinger and Schwartz 2005).

Genetically modified pigs with the inactivated GGTA1 gene facilitate the xenotransplantation of porcine tissues and organs into primates by preventing hyperacute rejection due to preexisting antibodies against the Gal antigen. Although pigs that lack Galα(1,3)Gal particles ensure prolonged survival of xenografts, other epitopes are still expressed and may induce acute humoral xenograft rejection as subsequent rejection. However, the response to non-Gal antigens is not as robust as the response against Gal antigens, the non-Gal antigens play a significant role and suppression of their production is required. Antibodies other than anti-Gal (anti-non-Gal antibodies) are found in the pre-transplant sera of most primates. In 2012, baboons were given hearts coming from pigs produced by crossing animals with the inactivated GGTA1 gene and expression of hCD46. The mean transplant survival time was 94 days, but the application of B lymphocyte depletion extended the maximum xenotrasplant survival in the organism of the recipient to 236 days. These results indicate a crucial role for B cells in the mechanisms of elicited anti-non-Gal antibodies and xenograft rejection. The efficient depletion of B cells was obtained by four doses of the anti-CD20 antibody (Mohiuddin et al. 2012). Another antigen occurring on the surface of porcine cells is cytidine monophosphate-N-acetylneuraminic acid hydroxylase (CMAH), which catalyzes the reactions forming the Neu5Gc antigen (N-glycolylneuraminic acid). Double transgenic pigs have already been produced with inactivated GGTA1 and CMAH genes. The lower cytotoxicity of human serum in comparison to peripheral blood mononuclear cells (PBMCs) coming from double transgenic pigs was shown in that study, in comparison to cells from animals with the inactivated GGTA1 gene (Lutz et al. 2013).

## Coagulation Barrier

Despite the long survival of α1,3-galactosyltransferase gene knockout (GTKO) and/or human CRP pig organs transplanted into non-human primates, thrombotic microangiopathy finally develops in the grafts (Kuwaki et al. 2005; Shimizu et al. 2008). It is characterized by fibrin deposition and platelet aggregation, which result in thrombosis in the vessels of the graft. This is probably associated with the numerous genetic differences between the recipient and the donor coagulation systems. The solution to thrombotic microangiopathy is to overexpress high levels of cell surface regulators. The main mechanisms that control the coagulation and propagation of thrombus are the tissue factor pathway inhibitor (TFPI) and the thrombomodulin (TBM)-protein C pathway. Failure of GTKO organ xenotransplantation provides evidence of thrombosis and platelet sequestration, and frequently development of consumptive coagulopathy in the primate recipients. Lin et al. (2010b) transplanted GTKO/CD46 pig kidneys into immunosuppressed baboons and reported that four recipients died or were euthanased between 9 and 16 days because of the development of consumptive coagulopathy. Consumptive coagulopathy have the features of intense thrombocytopenia, fibrinogen depletion and increased clotting times (Menoret et al. 2004). Expression of tissue factor (TF) on activated porcine endothelial cells induced by the immune response has been considered the major agent of consumptive coagulopathy. The coagulation response depends on the type of xenograft. Transcriptional assaying has suggested that renal xenografts may be more likely to initiate consumptive coagulopathy than cardiac xenografts (Knosalla et al. 2009). GTKO porcine hearts transplanted into baboons under immunosuppression were rejected after 59–179 days. Thrombotic microangiopathy developed, but none of the primate recipients developed consumptive coagulopathy (Shimizu et al. 2008). Ezzelarab et al. (2009) demonstrated that the majority of cardiac xenografts show features of thrombotic microangiopathy in the GTKO pig-to-baboon xenotransplantation model. Their results suggested that platelets are activated to express tissue factor soon after xenotransplantation, either in response to inflammatory factors or by contact with porcine endothelial cells (Lin et al. 2008).

Small genetic differences found between the recipient and the donor of the transplanted organ may lead to a disturbance in the equilibrium between pro- and anti-coagulant activity of the coagulation system. This subsequently leads to delayed xenograft rejection. Ectonucleoside triphosphate diphosphohydrolase-1 (CD39) is now recognized as a critical thromboregulatory protein (Kaczmarek et al. 1996). CD39 is an ectoenzyme found in the vascular endothelium and in blood cells, and plays a significant role in the regulation of coagulation and infection processes. Extracellular adenosine-triphosphate (ATP) and adenosine diphosphate (ADP) released at the site of cellular damage can induce platelet-mediated thrombosis. ADP is a potent platelet antagonist which recruits platelets to the site of injury, and enhances platelet aggregation. CD39 rapidly hydrolyzes ATP and ADP to adenosine monophosphate (AMP) and adenosine, which is a strong inhibitor of platelet aggregation. At the same time, AMP is hydrolyzed to adenosine by the ecto-5′-nucleotidase (CD73). The rapid reduction of CD39, resulting from endothelial cells activation and vascular injury, induces the activation and aggregation of platelets (Marcus et al. 2005). It was previously shown that the overexpression of human CD39 in mice prevents thrombosis, both in the case of transplantations of allogenic hearts in mice (Dwyer et al. 2004), and murine islets perfused with human blood (Dwyer et al. 2006), while it also protects transplanted murine kidneys against ischemia reperfusion injury (Crikis et al. 2010a). In the swine model, Wheeler et al. (2012) demonstrated that infarct size is significantly reduced following myocardial ischemia reperfusion injury in transgenic swine expressing human CD39. Moreover, Iwase at al. (2014) in their review described that initial studies of heart transplantation in baboons indicated that the transgenic expression of CD39 in the pig inhibited the early development of thrombocytopenia and reduction in fibrinogen. In comparison to human cells, endothelial cells in pigs have a markedly lower level of CD73, which reduces their capacity to produce adenosine causing blood coagulation (Khalpey et al. 2005). Expression of hCD39 should have a advantageous thromboregulatory result. However, it may also be a condition to express human CD73, which may increase this effect on the adenosine pathway.

The vascular endothelium produces the strongest natural inhibitor of the exogenous coagulation system, i.e. TFPI. The activity inhibiting the blood coagulation process by TFPI consists in the reversible inhibition of the Xa factor and formation of the Xa/TFPI complex, which subsequently inhibits the TF/VIIa complex. The TF inhibitor thus plays the role of a double TF inhibitor of the Xa factor in the coagulation process and the TF/VIIa complex. It is the only inhibitor acting in the preliminary phase of blood coagulation, preventing the formation of thrombin. The porcine TFPI is an ineffective inhibitor of the human Xa factor and may ineffectively shut the activation of the major TF (Cowan et al. 2009; Kopp et al. 1997). Overexpression of human TFPI has considerable potential to control the initiation of TF-dependent coagulation in xenotransplantation. The hearts of transgenic mice expressing human TFPI survived for a long time in immunosuppressed rats (Chen et al. 2004). Transgenic pigs produced using the same constructs showed lower expression of human TFPI, but it was sufficient to partially block anti-non-Gal IgG antibodies mediating in increased TF transcription (Lin et al. 2010a). Iwase et al. (2013) indicated that human platelet aggregation induced by swine aortic endothelial cells expressing human TFPI was significantly lower than that induced by wild-type.

Thrombomodulin is an integral membrane protein found on the surface of endothelial cells and participates in the inhibition of the coagulation process. After binding of thrombin participates in the activation of protein C, TBM acts as a strong anticoagulant. Protein C, together with the participation of protein S as a co-factor, inhibits the Va and VIIIa factors inactivating the enzymatic cascade leading to clot formation. Porcine TBM binds human thrombin less strongly and for this reason it does not activate protein C (Siegel et al. 1997; van’t Veer et al. 1997). Incorporating the human TBM gene into the graft donor genome would prevent inhibition of the activity of protein C involved in the process mentioned above. Expression of human TBM in transgenic mice resulted in the graft being protected without any negative effect on the health of animals in a mouse-to-rat model (Crikis et al. 2010b). Miwa et al. (2010) demonstrated that wild-type swine aortic endothelial cells transfected to express human TBM significantly suppressed prothrombinase activity and delayed human plasma clotting. Transgenic pigs were produced with the incorporated human TBM gene with a strong endothelium-specific expression in different organs, including the heart and kidneys (Aigner et al. 2010). However, the functional consequences have not been described to date. Moreover, Iwase et al. (2013) indicated that platelet aggregation induced by GTKO/CD46/TBM pig aortic endothelial cells was significantly lower than that induced by wild-type or GTKO.

The preeminent hurdles to xenotransplantation include thrombosis and inflammation. A potential solution may be the overexpression of the endothelial protein C receptor (EPCR) in donor pigs, which provides potent local anti-inflammatory, anticoagulant and cytoprotective cell signaling. One of the major mechanisms controlling the coagulation and propagation of thrombus is the TBM-protein C pathway. EPCR binds protein C on the endothelial cell surface, presents it to the TBM-thrombin complex and enhances the rate of protein C activation (Taylor et al. 2001). Activated protein C, together with co-factor protein S, down-regulates thrombin formation by inactivating factors Va and VIIIa, two important accelerators of clotting cascade (Esmon 2003, 2010). This entails a reduction of platelet activation and a decrease in thrombotic microangiopathy (Cerchiara et al. 2007). Lee et al. (2012) obtained transgenic mice expressing human EPCR (hEPCR), which were protected against warm renal ischemia reperfusion injury. Hearts from these transgenic mice demonstrated a significant prolongation of survival after heterotropic transplantation in rat recipients and showed less edema and hemorrhage. Iwase et al. (2013) also investigated the impact of pig genetic modifications (including hEPCR) on human platelet aggregation in response to pig aortic endothelial cells (pAEC) and indicated a significant positive correlation between a reduction in aggregation and EPCR expression on pAEC. Moreover, pig lungs expressing hEPCR demonstrated a prolonged functioning and decreased platelet activation in a xenogenic lung perfusion model (Burdorf et al. 2013). The results of these studies indicate that expression of hEPCR may provide protection against transplant-related thrombosis.

Porcine the von Willebrand factor (vWF) is a large multimeric glycoprotein which spontaneously aggregates human platelets even in the absence of sheer stress through an aberrant interaction between its O-glycosylated A1 domain and platelet glycoprotein Ib (GPIb) receptors (Schulte am et al. 2005). After platelet arrest due to the GPIb-vWF interaction, intracellular signaling occurs and platelets become activated. Once activated, circulating platelets are recruited to the place of endothelial cells injury and there they become a significant component in the development of thrombus (Gawaz 2004). The vWF plays a key role in the pathogenesis of xenograft failure, especially in pulmonary xenotransplantation because the lung releases more vWF than the heart or kidneys (Holzknecht et al. 2001). One of the solutions to these incompatibilities is to use pigs with von Willebrand disease characterized by a deficit of vWF in the blood. The use of pigs with von Willebrand disease in the orthotopic lung transplantation to baboons had no effect on the strong activation of coagulation. However, when pulmonary intravascular macrophages were inhibited, the total survival time improved from 5 to almost 100 h with a delay in the onset of coagulopathy (Cantu et al. 2007). Another potential solution is connected with the fact that the level of O-glycosylation of the A1 domain in the vWF of pigs regulates the degree of human platelet activation (Schulte am et al. 2005). A decrease in vWF activity in pigs may be observed in the elimination of the primary glycosylation enzyme as in GTKO pigs. Improved compatibility remains difficult to measure, because thrombotic microangiopathy is not eliminated in GTKO transplantations (Knosalla et al. 2009; Shimizu et al. 2008).

## NK Cells Cytotoxicity

Activated NK cells are capable of destruction of the xenotransplant cells, having a cytotoxic effect either directly or through antibodies in the antibody-dependent cellular cytotoxicity (ADCC) (Baumann et al. 2004; Kitchens et al. 2006; Rieben and Seebach 2005). To kill the target cell in ADCC, both antibodies coating the target cell and determining reaction specificity and effector cells determining the cytotoxic effect need to be involved simultaneously. Inactivation of the GGTA1 gene limits ADCC, although it does not protect against direct cytotoxicity of NK cells (Baumann et al. 2004). The direct cytotoxic effect of NK cells is regulated by two types of opposite signals: activating and inhibitory, transferred by respective receptors found on the surface of these cells (Lopez-Botet and Bellon 1999). Through activating receptors, lytic mechanisms are induced, leading to the death of the target cell. In turn, the inhibitory receptor interacting with a respective molecule on the target cell leads to the cytotoxic reaction being blocked. Depending on which signals predominate, the target cell is either eliminated or saved. Thus it is assumed that elimination of ligands for receptors activating NK cells on porcine cells or increasing the level of ligands for inhibitory receptors might prevent xenograft rejection involving NK cells.

The susceptibility of porcine endothelial cells to lysis caused by human NK cells is caused, among other things, by the inability to recognize porcine major histocompatibility complex (MHC) class I molecules by inhibitory receptors of human NK cells. This results from differences within the MHC of pigs (swine leukocyte antigens) and that of humans (human leukocyte antigens, HLA) (Sullivan et al. 1997). In this context HLA-E molecules are potential activators of NK inhibitory receptors (CD94/NKG2A). Bongoni et al. (2014) examined the potential of combined overexpression of hCD46 and HLA-E in an ex vivo pig-to-human xenoperfusion model. Double transgenic hCD46/HLA-E expression evidently reduced four factors: humoral responses, the terminal pathway of complement activation, endothelial cell activation and inflammatory cytokine production. Moreover, Maeda et al. (2013) indicate that generating transgenic HLA-E pigs might protect porcine grafts not only from NK cytotoxicity, but also from macrophage-mediated cytotoxicity. HLA-E is accumulated in the endoplasmic reticulum and is transported to the cell surface as a stable trimeric complex consisting of the HLA-E heavy chain, β2 microglobulin and a peptide derived from the leader sequence of other MHC class I molecules (Ulbrecht et al. 1999). Surface expression of HLA-E, specific binding to the CD94/NKG2 dimer and prevention of NK-related lysis all depend on the nature of the peptide included in the HLA-E trimeric complex (Miller et al. 2003). To use this mechanism, transgenic pigs were generated with the HLA-E gene along with the signal sequence and human β2 microglobulin (Weiss et al. 2009). Lymphoblasts and endothelial cells coming from these transgenic pigs were partly protected against the cytotoxicity of human NK cells (Weiss et al. 2009). However, expression of HLA-E influences neither the adhesion of human NK cells to porcine endothelial cells, nor the formation of bonds between human NK cells and porcine cells (Lilienfeld et al. 2007). Thus it is suggested that effective prevention of NK-dependent xenograft rejection requires a simultaneous application of several protective strategies.

Partial protection against the cytotoxicity of human NK cells is also provided by the expression of HLA-Cw3, HLA-Cw4 or HLA-G genes in porcine cells (Forte et al. 2000, 2001, 2009; Seebach et al. 1997; Sharland et al. 2002). Migration of NK cells through the vessel walls occurs in several successive stages: NK cells rolling over the endothelium, activation, firm adhesion and transmigration. Expression of HLA-G in porcine cells also disturbs the rolling of NK cells over the endothelium and transmigration of NK cells between porcine endothelial cells (Dorling et al. 2000; Forte et al. 2001). To date it has not been established whether HLA-G expression in porcine cells may be functionally optimized by expression of human β2 microglobulin, as was described for HLA-E (Crew et al. 2005).

The cytotoxic effect on porcine endothelial cells leading to their lysis is also caused by binding with the NKG2D receptors of the porcine ULBP1 antigen (UL-16 binding protein 1), as well as the currently unidentified antigen with the NKp44 receptor (Forte et al. 2005; Lilienfeld et al. 2006, 2008; Lin et al. 1997; Matter-Reissmann et al. 2002; Tran et al. 2008; Tsuyuki et al. 2001). Forte et al. (2005) showed that lysis of porcine endothelial cells, resulting from the presence of the freshly isolated human NK cells, is mainly caused by NKG2D. On the other hand, lysis of porcine endothelial cells caused by human NK cells activated by interleukin (IL)-2 (this cytokine enhances cytotoxic activity) depends on both receptors, i.e. NKG2D and NKp44. Lilienfeld et al. (2006) demonstrated that cytotoxicity targeting porcine endothelial cells, which is caused both by the freshly isolated NK cells and IL-2-activated NK cells through the NKG2D receptor, was completely blocked using anti- pULBP1 polyclonal antibody. This suggests that porcine ULBP1 is the primary functional ligand in pigs for human NKG2D. For this reason eliminating porcine ULBP1 from pig cells is the next extremely important step towards protecting pig-origin xenotransplants against the cytotoxicity of human NK cells.

## Macrophages in Xenograft Rejection

Macrophages participate in all the three types of xenograft rejection, both hyperacute rejection, xenograft rejection and cellular xenograft rejection (Candinas et al. 1996; Lin et al. 1997; Wallgren et al. 1995). Their activation may result from the activity of xenoreactive T lymphocytes or direct interactions of antigens from graft donor endothelial cells with receptors found on the surface of macrophages (Yi et al. 2003). It was shown that macrophages in primates mediate xenograft rejection of porcine pancreatic islets with removed Gal antigens and cause direct rejection of xenogenic bone marrow cells even in the absence of adaptive immunity. This constitutes a tremendous obstacle in the application of mixed chimerism to induce tolerance towards a xenogenic graft (Basker et al. 2001; McKenzie et al. 1994; Tseng et al. 2005b). Moreover, Ide et al. (2005) showed that human macrophages phagocyte porcine red blood cells in a manner independent of antibodies or the complement, and the removal of the Gal epitope from porcine cells did not prevent their phagocytosis.

The factor limiting phagocytosis is the CD47 (also known as integrin-associated protein) surface antigen (Oldenborg et al. 2000). CD47 belongs to the Ig superfamily and is expressed in all tissues. Signaling regulatory protein (SIRPα also known as SHPS-1, BIT, CD172a or p84) belongs to the family of membrane proteins and is an inhibitory receptor expressed on macrophages and dendritic cells, which recognizes CD47 as a “marker of self” (Barclay and Brown 2006; Brown and Frazier 2001; Kharitonenkov et al. 1997; Seiffert et al. 1999). The interaction of CD47-SIRPα is a “don’t eat me” signal to macrophages, and in this way inhibits phagocytosis. The CD47 factor found on the surface of endothelial cells in pigs differs from its equivalent found on the surface of human endothelial cells. Although Subramanian et al. (2006) reported that human SIRPα may bind to porcine CD47, Ide et al. (2007) showed that binding porcine CD47 does not supply the inhibiting signal via SIRPα to human macrophages. The same research team also demonstrated that expression of human CD47 in pig cells reduced susceptibility to phagocytosis by human macrophages in vitro. Recently, Tena et al. (2013) produced a GTKO miniature pig with expression of human CD47 to obtain the source animal for future swine-to-baboon bone marrow transplant experiments.

## Immunosuppressive Agents

Immunosuppressive agents are commonly used to prevent allograft rejection, but also in experiments on pig organ xenotransplantation models. Immunosuppressive regimens should do the following: extend xenograft survival, minimize the side-effects of the immunosuppression and make clinical trials possible. The most common immunosuppressive agents used in xenotransplantation are the anti-CD154 antibody, mycophenolate mofetil, anti-thymocyte globulin, tacrolimus, rapamycin, cyclosporine, belatacept, abatacept, sirolimus, fingolimod and everolimus (Ekser et al. 2011; Graham and Schuurman 2013). Owing to immunosuppressive regimens, survival after heterotopic heart xenotransplantation now extends to 8 months (Mohiuddin et al. 2012) and after life-supporting kidney xenotransplantation to nearly 3 months (Griesemer et al. 2009).

Cyclosporine and tacrolimus resulted in increase in platelet aggregation via the serotonin pathway. Cyclosporine diminishes the activity of the immune system by inhibits T-helper cell production of growth factors important for B cell and cytotoxic T-cell differentiation and proliferation, while allowing expansion of suppressor T-cell populations. Tacrolimus inhibits T-lymphocyte activation, blocks activation of nitric oxide synthetase, and potentiates the cellular effects of steroids. Anti-thymocyte globulin is an infusion of rabbit-derived antibodies against human T cells, which is used in the prevention and treatment of acute rejection in organ transplantation. Rabbit anti-thymocyte globulin effects large reductions in the number of circulating T lymphocytes, hence preventing the cellular rejection of transplanted organs. Sirolimus prevents activation of T cells and B cells by inhibiting their response to IL-2. Everolimus is a inhibitor of mammalian target of rapamycin that is structurally similar to sirolimus, but with a number of important pharmacokinetic differences, including a shorter half-life and time to steady state. Belatacept is a protein composed of the Fc fragment of a human IgG1 immunoglobulin linked to the extracellular domain of cytotoxic T-lymphocyte antigen 4, which is a molecule important for T-cell co-stimulation, blocking the process of T-cell activation. Mycophenolate mofetil inhibits proliferation of T and B lymphocytes by blocking the production of guanosine nucleotides required for DNA synthesis. Abatacept is a selective costimulation modulator as it inhibits the costimulation of T cells. Whereas fingolimod is a sphingosine 1-phosphate receptor modulator, which sequesters lymphocytes in lymph nodes, preventing them from contributing to an autoimmune reaction. CD154 is a protein that is mainly expressed on activated T cells and is a member of the TNF superfamily of molecules. Ezzelarab et al. (2009) indicated that an immunosuppression regimen including costimulation blockade with an anti-CD154 monoclonal antibody prolonged graft survival, but xenograft failure was associated with activation of the innate immune system. Consumptive coagulopathy occurred in six of nine recipients. Similarly, Mohiuddin et al. (2012) demonstrated that costimulation blockade via anti-CD154 antibody significantly prolongs the cardiac xenograft survival in a GTKO/CD46 pig-to-baboon heterotopic xenotransplantation model. However, many coagulation disorders were observed with the use of anti-CD154 antibody, and recipient survival was markedly reduced by these complications.

## Porcine Endogenous Retroviruses

The transplantation of porcine organs, tissues, or cells is associated with the potential risk of various pathogen infections. Most of the potential porcine zoonotic microorganisms can be eliminated by careful screening and qualified pathogen-free breeding. Only porcine endogenous retroviruses (PERVs) permanently integrated into the genome of pigs represent a risk. PERV-A and PERV-B are probably present in all pigs and are able to infect human cells in vitro (Takeuchi et al. 1998; Wilson et al. 2000). PERV-C only infects pig cells and is not present in all pigs (Takeuchi et al. 1998). However, recombination between PERV-A and PERV-C may result in a high-titer PERV-A/C strain that is able to infect human cells (Denner 2008). The International Xenotransplantation Association specified the conditions which must be established in clinical trials with pig cells. They are as follows: careful screening for PERVs in the source pig herd, selection of pigs which exhibit a low-level expression of PERV-A and PERV-B, selection of pigs which do not contain PERV-C in their germ line and screening of xenotransplant recipients for PERV transmission (Denner et al. 2009). Additionally, RNA interference technology can be used to inhibit expression of PERV genes in transgenic pigs. Efficient gene-specific small interfering RNAs corresponding to highly conserved regions in the *pol* gene of the PERV genome were selected and were able to inhibit expression of all PERV subtypes in PERV-infected human cells as well as in primary pig cells (Karlas et al. 2004).

In contrast to in vitro infection studies, there is no evidence of any pig viruses being transmitted into nonhuman primate recipients (Bauer et al. 2010; Garkavenko et al. 2008; Loss et al. 2001; Martin et al. 1998; Moscoso et al. 2005; Nishitai et al. 2005; Simon et al. 2003; Specke et al. 2002, 2009; Wilson et al. 2000). However, pig-to-nonhuman-primate transplant models to study cross-species PERV transmission have several limitations, including differences in viral receptors. A mutation in the PERV receptor in some nonhuman primates significantly reduces the efficiency of infection, and the predictive value of this model system remains unclear.

What is more, there is no evidence of PERV transmission in clinical trials either. Vital pig skin has been widely used as a dressing for clinical treatment for burn injuries. Scobie et al. (2013) investigated the presence of PERVs by analyzing the blood of burn patients who had received living pig-skin dressings for up to 8 weeks. No PERV genomic material or anti-PERV antiobdy response was found. There was no evidence of infection with PERV or other porcine microorganisms in recipients of pig islet transplants for the treatment of diabetes (Elliott et al. 2000; Garkavenko et al. 2004). Furthermore, no evidence was found of PERV provirus integration in the DNA from PBMCs of patients who received porcine fetal neuronal cells for treatment of Parkinson’s disease, Huntington’s disease, and epilepsy (Dinsmore et al. 2000; Fink et al. 2000; Schumacher et al. 2000).

## Immunological Tolerance

Several problems connected with graft rejection might be solved by the induction of immune tolerance. In relation to organ transplantations tolerance is the condition of no response to graft antigens with no need for long-term immunosuppression treatment. In the case of xenotransplantation, two approaches are proposed to achieve specific tolerance. One of them is to induce tolerance by the generation of molecular chimerism. This method consists in the autologous transplantation of bone marrow, whose cells contain the previously incorporated porcine GGTA1 gene. Production of the Gal antigen by the genetically altered cells induces tolerance to this antigen. The other approach is to produce chimerism of hematopoietic cells and by pig thymus transplantation (Griesemer et al. 2010; Tseng et al. 2005b; Yamada et al. 2005).

In summary, for successfully introducing xenotransplantation into the clinic, further multiple genetic modifications pigs (to overcome innate, coagulopathic and inflammatory responses) and more effective immunosuppressive and adjunctive regimens are required.

## References

[CR1] Aigner B, Klymiuk N, Wolf E (2010). Transgenic pigs for xenotransplantation: selection of promoter sequences for reliable transgene expression. Curr Opin Organ Transplant.

[CR2] Artrip JH, Kwiatkowski P, Michler RE (1999). Target cell susceptibility to lysis by human natural killer cells is augmented by alpha(1,3)-galactosyltransferase and reduced by alpha(1,2)-fucosyltransferase. J Biol Chem.

[CR3] Barclay AN, Brown MH (2006). The SIRP family of receptors and immune regulation. Nat Rev Immunol.

[CR4] Basker M, Alwayn IP, Buhler L (2001). Clearance of mobilized porcine peripheral blood progenitor cells is delayed by depletion of the phagocytic reticuloendothelial system in baboons. Transplantation.

[CR5] Bauer A, Postrach J, Thomann M (2010). First experience with heterotopic thoracic pig-tobaboon cardiac xenotransplantation. Xenotransplantation.

[CR6] Baumann BC, Forte P, Hawley RJ (2004). Lack of galactose-alpha-1,3-galactose expression on porcine endothelial cells prevents complement-induced lysis but not direct xenogeneic NK cytotoxicity. J Immunol.

[CR7] Bongoni AK, Kiermeir D, Jenni H (2014). Complement dependent early immunological responses during ex vivo xenoperfusion of hCD46/HLA-E double transgenic pig forelimbs with human blood. Xenotransplantation.

[CR8] Brown EJ, Frazier WA (2001). Integrin-associated protein (CD47) and its ligands. Trends Cell Biol.

[CR9] Burdorf L, Rybak E, Zhang T (2013). Human EPCR expression in GalTKO.hCD46 lungs extends survival time and lowers PVR in a xenogenic lung perfusion model. J Heart Lung Transplant.

[CR10] Byrne GW, Davies WR, Oi K (2006). Increased immunosuppression, not anticoagulation, extends cardiac xenograft survival. Transplantation.

[CR11] Candinas D, Belliveau S, Koyamada N (1996). T cell independence of macrophage and natural killer cell infiltration, cytokine production, and endothelial activation during delayed xenograft rejection. Transplantation.

[CR12] Cantu E, Balsara KR, Li B (2007). Prolonged function of macrophage, von Willebrand factor-deficient porcine pulmonary xenografts. Am J Transplant.

[CR13] Cerchiara E, Tirindelli MC, Giannetti B (2007). The numerous properties of the anticoagulant protein C. Clin Ter.

[CR14] Chen RH, Naficy S, Logan JS (1999). Hearts from transgenic pigs constructed with CD59/DAF genomic clones demonstrate improved survival in primates. Xenotransplantation.

[CR15] Chen D, Weber M, McVey JH (2004). Complete inhibition of acute humoral rejection using regulated expression of membrane-tethered anticoagulants on xenograft endothelium. Am J Transplant.

[CR16] Cooper DK, Gollackner B, Sachs DH (2002). Will the pig solve the transplantation backlog?. Annu Rev Med.

[CR17] Costa C, Zhao L, Burton WV (1999). Expression of the human alpha1,2-fucosyltransferase in transgenic pigs modifies the cell surface carbohydrate phenotype and confers resistance to human serum-mediated cytolysis. FASEB J.

[CR18] Cowan PJ, Roussel JC, d’Apice AJ (2009). The vascular and coagulation issues in xenotransplantation. Curr Opin Organ Transplant.

[CR19] Cozzi E, Bhatti F, Schmoeckel M (2000). Long-term survival of nonhuman primates receiving life-supporting transgenic porcine kidney xenografts. Transplantation.

[CR20] Crew MD, Cannon MJ, Phanavanh B (2005). An HLA-E single trimer inhibits human NK cell reactivity towards porcine cells. Mol Immunol.

[CR21] Crikis S, Lu B, Murray-Segal LM (2010). Transgenic overexpression of CD39 protects against renal ischemia-reperfusion and transplant vascular injury. Am J Transplant.

[CR22] Crikis S, Zhang XM, Dezfouli S (2010). Anti-inflammatory and anticoagulant effects of transgenic expression of human thrombomodulin in mice. Am J Transplant.

[CR23] Dai Y, Vaught TD, Boone J (2002). Targeted disruption of the alpha1,3-galactosyltransferase gene in cloned pigs. Nat Biotechnol.

[CR24] Daniels LJ, Platt JL (1997). Hyperacute xenograft rejection as an immunologic barrier to xenotransplantation. Kidney Int Suppl.

[CR25] Denner J (2008). Recombinant porcine endogenous retroviruses (PERVA/C): a new risk for xenotransplantation?. Arch Virol.

[CR26] Denner J, Schuurman HJ, Patience C (2009). The International Xenotransplantation Association consensus statement on conditions for undertaking clinical trials of porcine islet products in type 1 diabetes—chapter 5: strategies to prevent transmission of porcine endogenous retroviruses. Xenotransplantation.

[CR27] Deppenmeier S, Bock O, Mengel M (2006). Health status of transgenic pigs expressing the human complement regulatory protein CD59. Xenotransplantation.

[CR28] Diamond LE, McCurry KR, Martin MJ (1996). Characterization of transgenic pigs expressing functionally active human CD59 on cardiac endothelium. Transplantation.

[CR29] Dinsmore JH, Manhart C, Raineri R (2000). No evidence for infection of human cells with porcine endogenous retrovirus (PERV) after exposure to porcine fetal neuronal cells. Transplantation.

[CR30] Dorling A, Monk NJ, Lechler RI (2000). HLA-G inhibits the transendothelial migration of human NK cells. Eur J Immunol.

[CR31] Dwyer KM, Robson SC, Nandurkar HH (2004). Thromboregulatory manifestations in human CD39 transgenic mice and the implications for thrombotic disease and transplantation. J Clin Invest.

[CR32] Dwyer KM, Mysore TB, Crikis S (2006). The transgenic expression of human CD39 on murine islets inhibits clotting of human blood. Transplantation.

[CR33] Ekser B, Kumar G, Veroux M (2011). Therapeutic issues in the treatment of vascularized xenotransplants using gal-knockout donors in nonhuman primates. Curr Opin Organ Transplant.

[CR34] Elliott RB, Escobar L, Garkavenko O (2000). No evidence of infection with porcine endogenous retrovirus in recipients of encapsulated porcine islet xenografts. Cell Transplant.

[CR35] Esmon CT (2003). The protein C pathway. Chest.

[CR36] Esmon CT (2010). The discovery of the endothelial cell protein C receptor. J Thromb Haemost.

[CR37] Ezzelarab M, Garcia B, Azimzadeh A (2009). The innate immune response and activation of coagulation in alpha1,3-galactosyltransferase gene-knockout xenograft recipients. Transplantation.

[CR38] Fink JS, Schumacher JM, Ellias SL (2000). Porcine xenografts in Parkinson’s disease and Huntington’s disease patients: preliminary results. Cell Transplant.

[CR39] Fodor WL, Williams BL, Matis LA (1994). Expression of a functional human complement inhibitor in a transgenic pig as a model for the prevention of xenogeneic hyperacute organ rejection. Proc Natl Acad Sci USA.

[CR40] Forte P, Matter-Reissmann UB, Strasser M (2000). Porcine aortic endothelial cells transfected with HLA-G are partially protected from xenogeneic human NK cytotoxicity. Hum Immunol.

[CR41] Forte P, Pazmany L, Matter-Reissmann UB (2001). HLA-G inhibits rolling adhesion of activated human NK cells on porcine endothelial cells. J Immunol.

[CR42] Forte P, Lilienfeld BG, Baumann BC (2005). Human NK cytotoxicity against porcine cells is triggered by NKp44 and NKG2D. J Immunol.

[CR43] Forte P, Baumann BC, Schneider MK (2009). HLA-Cw4 expression on porcine endothelial cells reduces cytotoxicity and adhesion mediated by CD158a + human NK cells. Xenotransplantation.

[CR44] Galili U, Shohet SB, Kobrin E (1988). Man, apes, and Old World monkeys differ from other mammals in the expression of alpha-galactosyl epitopes on nucleated cells. J Biol Chem.

[CR45] Garkavenko O, Croxson MC, Irgang M (2004). Monitoring for presence of potentially xenotic viruses in recipients of pig islet xenotransplantation. J Clin Microbiol.

[CR46] Garkavenko O, Dieckhoff B, Wynyard S (2008). Absence of transmission of potentially xenotic viruses in a prospective pig to primate islet xenotransplantation study. J Med Virol.

[CR47] Gawaz M (2004). Role of platelets in coronary thrombosis and reperfusion of ischemic myocardium. Cardiovasc Res.

[CR48] Graham ML, Schuurman HJ (2013). The usefulness and limitations of the diabetic macaque model in evaluating long-term porcine islet xenograft survival. Xenotransplantation.

[CR49] Griesemer AD, Hirakata A, Shimizu A (2009). Results of gal-knockout porcine thymokidney xenografts. Am J Transplant.

[CR50] Griesemer AD, Sorenson EC, Hardy MA (2010). The role of the thymus in tolerance. Transplantation.

[CR51] Holzknecht ZE, Coombes S, Blocher BA (2001). Immune complex formation after xenotransplantation: evidence of type III as well as type II immune reactions provide clues to pathophysiology. Am J Pathol.

[CR52] Ide K, Ohdan H, Kobayashi T (2005). Antibody- and complement-independent phagocytotic and cytolytic activities of human macrophages toward porcine cells. Xenotransplantation.

[CR53] Ide K, Wang H, Tahara H (2007). Role for CD47-SIRPalpha signaling in xenograft rejection by macrophages. Proc Natl Acad Sci USA.

[CR54] Ingelfinger JR, Schwartz RS (2005). Immunosuppression-the promise of specificity. N Engl J Med.

[CR55] Iwase H, Ekser B, Hara H (2013). Regulation of human platelet aggregation by genetically modified pig endothelial cells and thrombin inhibition. Xenotransplantation.

[CR56] Iwase H, Ezzelarab MB, Ekser B (2014). The role of platelets in coagulation dysfunction in xenotransplantation and therapeutic options. Xenotransplantation.

[CR57] Jia Y, Ren H, Gao X (2004). Expression of human alpha-galactosidase and alpha1,2-fucosyltransferase genes modifies the cell surface Galalpha1,3Gal antigen and confers resistance to human serum-mediated cytolysis. Chin Med Sci J.

[CR58] Kaczmarek E, Koziak K, Sévigny J (1996). Identification and characterization of CD39/vascular ATP diphosphohydrolase. J Biol Chem.

[CR59] Karlas A, Kurth R, Denner J (2004). Inhibition of porcine endogenous retroviruses by RNA interference: increasing the safety of xenotransplantation. Virology.

[CR60] Khalpey Z, Yuen AH, Kalsi KK (2005). Loss of ecto-5’-nucleotidase from porcine endothelial cells after exposure to human blood: Implications for xenotransplantation. Biochim Biophys Acta.

[CR61] Kharitonenkov A, Chen Z, Sures I (1997). A family of proteins that inhibit signalling through tyrosine kinase receptors. Nature.

[CR62] Kitchens WH, Uehara S, Chase CM (2006). The changing role of natural killer cells in solid organ rejection and tolerance. Transplantation.

[CR63] Knosalla C, Yazawa K, Behdad A (2009). Renal and cardiac endothelial heterogeneity impact acute vascular rejection in pig-to baboon xenotransplantation. Am J Transplant.

[CR64] Kopp CW, Siegel JB, Hancock WW (1997). Effect of porcine endothelial tissue factor pathway inhibitor on human coagulation factors. Transplantation.

[CR65] Kuwaki K, Tseng YL, Dor FJ (2005). Heart transplantation in baboons using alpha1,3-galactosyltransferase gene-knockout pigs as donors: initial experience. Nat Med.

[CR66] Kwiatkowski P, Artrip JH, John R (1999). Induction of swine major histocompatibility complex class I molecules on porcine endothelium by tumor necrosis factor-alpha reduces lysis by human natural killer cells. Transplantation.

[CR67] Lai L, Kolber-Simonds D, Park KW (2002). Production of alpha-1,3-galactosyltransferase knockout pigs by nuclear transfer cloning. Science.

[CR68] Langford GA, Yannoutsos N, Cozzi E (1994). Production of pigs transgenic for human decay accelerating factor. Transplant Proc.

[CR69] Larsen RD, Ernst LK, Nair RP (1990). Molecular cloning, sequence, and expression of a human GDP-l-fucose: beta-d-galactoside 2-alpha-l-fucosyltransferase cDNA that can form the H blood group antigen. Proc Natl Acad Sci USA.

[CR70] Lee KF, Lu B, Roussel JC (2012). Protective effects of transgenic human endothelial protein C receptor expression in murine models of transplantation. Am J Transplant.

[CR71] Lilienfeld BG, Garcia-Borges C, Crew MD (2006). Porcine UL16-binding protein 1 expressed on the surface of endothelial cells triggers human NK cytotoxicity through NKG2D. J Immunol.

[CR72] Lilienfeld BG, Crew MD, Forte P (2007). Transgenic expression of HLA-E single chain trimer protects porcine endothelial cells against human natural killer cellmediated cytotoxicity. Xenotransplantation.

[CR73] Lilienfeld BG, Schildknecht A, Imbach LL (2008). Characterization of porcine UL16-binding protein 1 endothelial cell surface expression. Xenotransplantation.

[CR74] Lin Y, Vandeputte M, Waer M (1997). Contribution of activated macrophages to the process of delayed xenograft rejection. Transplantation.

[CR75] Lin CC, Chen D, Mcvey JH (2008). Expression of tissue factor and initiation of clotting by human platelets and monocytes after incubation with porcine endothelial cells. Transplantation.

[CR76] Lin CC, Ezzelarab M, Hara H (2010). Atorvastatin or transgenic expression of TFPI inhibits coagulation initiated by anti-nonGal IgG binding to porcine aortic endothelial cells. J Thromb Haemost.

[CR77] Lin CC, Ezzelarab M, Shapiro R (2010). Tissue factor expression on recipient platelets is associated with consumptive coagulopathy in pig-to-primate kidney xenotransplantation. Am J Transplant.

[CR78] Lopez-Botet M, Bellon T (1999). Natural killer cell activation and inhibition by receptors for MHC class I. Curr Opin Immunol.

[CR79] Loss M, Arends H, Winkler M (2001). Analysis of potential porcine endogenous retrovirus (PERV) transmission in a whole-organ xenotransplantation model without interfering microchimerism. Transpl Int.

[CR80] Lutz AJ, Li P, Estrada JL (2013). Double knockout pigs deficient in *N*-glycolylneuraminic acid and galactose α-1,3-galactose reduce the humoral barrier to xenotransplantation. Xenotransplantation.

[CR81] Maeda A, Kawamura T, Ueno T (2013). The suppression of inflammatory macrophage-mediated cytotoxicity and proinflammatory cytokine production by transgenic expression of HLA-E. Transpl Immunol.

[CR82] Marcus AJ, Broekman MJ, Drosopoulos JH (2005). Role of CD39 (NTPDase-1) in thromboregulation, cerebroprotection, and cardioprotection. Semin Thromb Hemost.

[CR83] Martin U, Steinhoff G, Kiessig V (1998). Porcine endogenous retrovirus (PERV) was not transmitted from transplanted porcine endothelial cells to baboons in vivo. Transpl Int.

[CR84] Matter-Reissmann UB, Forte P, Schneider MK (2002). Xenogeneic human NK cytotoxicity against porcine endothelial cells is perforin/granzyme B dependent and not inhibited by Bcl-2 overexpression. Xenotransplantation.

[CR85] McGregor CG, Davies WR, Oi K (2005). Cardiac xenotransplantation: recent preclinical progress with 3-month median survival. J Thorac Cardiovasc Surg.

[CR86] McKenzie IF, Xing PX, Vaughan HA (1994). Distribution of the major xenoantigen (gal (alpha-1-3)gal) for pig to human xenografts. Transpl Immunol.

[CR87] Menoret S, Plat M, Blancho G (2004). Characterization of human CD55 and CD59 transgenic pigs and kidney xenotransplantation in the pig-to-baboon combination. Transplantation.

[CR88] Miller JD, Weber DA, Ibegbu C (2003). Analysis of HLA-E peptide-binding specificity and contact residues in bound peptide required for recognition by CD94/NKG2. J Immunol.

[CR89] Miwa Y, Yamamoto K, Onishi A (2010). Potential value of human thrombomodulin and DAF expression for coagulation control in pig-to-human xenotransplantation. Xenotransplantation.

[CR90] Mohiuddin MM, Corcoran PC, Singh AK (2012). B-cell depletion extends the survival of GTKO.hCD46Tg pig heart xenografts in baboons for up to 8 months. Am J Transplant.

[CR91] Moscoso I, Hermida-Prieto M, Mañez R (2005). Lack of cross-species transmission of porcine endogenous retrovirus in pig-to-baboon xenotransplantation with sustained depletion of anti-alphagal antibodies. Transplantation.

[CR92] Mulligan MS, Shearon TH, Weill D (2008). Heart and lung transplantation in the United States, 1997–2006. Am J Transplant.

[CR93] Niemann H, Verhoeyen E, Wonigeit K (2001). Cytomegalovirus early promoter induced expression of hCD59 in porcine organs provides protection against hyperacute rejection. Transplantation.

[CR94] Nishitai R, Ikai I, Shiotani T (2005). Absence of PERV infection in baboons after transgenic porcine liver perfusion. J Surg Res.

[CR95] Oldenborg PA, Zheleznyak A, Fang YF (2000). Role of CD47 as a marker of self on red blood cells. Science.

[CR96] Osman N, McKenzie IF, Ostenried K (1997). Combined transgenic expression of alpha-galactosidase and alpha1,2-fucosyltransferase leads to optimal reduction in the major xenoepitope Galalpha(1,3)Gal. Proc Natl Acad Sci USA.

[CR97] Phelps CJ, Koike C, Vaught TD (2003). Production of alpha 1,3-galactosyltransferase-deficient pigs. Science.

[CR98] Ramsoondar JJ, Machaty Z, Costa C (2003). Production of alpha 1,3-galactosyltransferase-knockout cloned pigs expressing human alpha 1,2-fucosylosyltransferase. Biol Reprod.

[CR99] Rieben R, Seebach JD (2005). Xenograft rejection: IgG1, complement and NK cells team up to activate and destroy the endothelium. Trends Immunol.

[CR100] Sandrin MS, McKenzie IF (1994). Gal alpha (1,3)Gal, The major xenoantigen(s) recognised in pigs by human natural antibodies. Immunol Rev.

[CR101] Schulte am EJ, Robson SC, Knoefel WT (2005). Olinked glycosylation and functional incompatibility of porcine von Willebrand factor for human platelet GPIb receptors. Xenotransplantation.

[CR102] Schumacher JM, Ellias SA, Palmer EP (2000). Transplantation of embryonic porcine mesencephalic tissue in patients with PD. Neurology.

[CR103] Scobie L, Padler-Karavani V, Le Bas-Bernardet S (2013). Long-term IgG response to porcine Neu5Gc antigens without transmission of PERV in burn patients treated with porcine skin xenografts. J Immunol.

[CR104] Seebach JD, Comrack C, Germana S (1997). HLA-Cw3 expression on porcine endothelial cells protects against xenogeneic cytotoxicity mediated by a subset of human NK cells. J Immunol.

[CR105] Seiffert M, Cant C, Chen Z (1999). Human signal-regulatory protein is expressed on normal, but not on subsets of leukemic myeloid cells and mediates cellular adhesion involving its counterreceptor CD47. Blood.

[CR106] Sharland A, Patel A, Lee JH (2002). Genetically modified HLA class I molecules able to inhibit human NK cells without provoking alloreactive CD8 + CTLs. J Immunol.

[CR107] Sharma A, Okabe J, Birch P (1996). Reduction in the level of Gal(alpha1,3)Gal in transgenic mice and pigs by the expression of an alpha(1,2)fucosyltransferase. Proc Natl Acad Sci USA.

[CR108] Sharma A, Naziruddin B, Cui C (2003). Pig cells that lack the gene for alpha1-3 galactosyltransferase express low levels of the gal antigen. Transplantation.

[CR109] Shimizu A, Hisashi Y, Kuwaki K (2008). Thrombotic microangiopathy associated with humoral rejection of cardiac xenografts from alpha1,3-galactosyltransferase gene-knockout pigs in baboons. Am J Pathol.

[CR110] Siegel JB, Grey ST, Lesnikoski BA (1997). Xenogeneic endothelial cells activate human prothrombin. Transplantation.

[CR111] Simon AR, Templin C, Schröder C (2003). No evidence for productive PERV infection of baboon cells in in vivo infection model. Ann Transplant.

[CR112] Specke V, Schuurman HJ, Plesker R (2002). Virus safety in xenotransplantation: preliminary data from first small animal and non-human primate in vivo experiments. Transplant Immunol.

[CR113] Specke V, Plesker R, Wood J (2009). No in vivo infection of triple immunosuppressed non-human primates after inoculation with high titers of porcine endogenous retroviruses. Xenotransplantation.

[CR114] Subramanian S, Parthasarathy R, Sen S (2006). Species- and cell type-specific interactions between CD47 and human SIRPalpha. Blood.

[CR115] Sullivan JA, Oettinger HF, Sachs DH (1997). Analysis of polymorphism in porcine MHC class I genes: alterations in signals recognized by human cytotoxic lymphocytes. J Immunol.

[CR116] Takeuchi Y, Patience C, Magre S (1998). Host range and interference studies of three classes of pig endogenous retrovirus. J Virol.

[CR117] Taylor FB, Peer GT, Lockhart MS (2001). Endothelial cell protein C receptor plays an important role in protein C activation in vivo. Blood.

[CR118] Tena A, Leonard D, Tasaki M (2013). Initial evidence for functional immune modulation in primate recipients of porcine skin grafts following conditioning with human CD47 transgeneic pig hematopoietic stem cells. Xenotransplantation.

[CR119] Tran PD, Christiansen D, Winterhalter A (2008). Porcine cells express more than one functional ligand for the human lymphocyte activating receptor NKG2D. Xenotransplantation.

[CR120] Tseng YL, Kuwaki K, Dor FJ (2005). Alpha1,3-Galactosyltransferase gene-knockout pig heart transplantation in baboons with survival approaching 6 months. Transplantation.

[CR121] Tseng YL, Sachs DH, Cooper DK (2005). Porcine hematopoietic progenitor cell transplantation in nonhuman primates: a review of progress. Transplantation.

[CR122] Tsuyuki S, Horvath-Arcidiacono JA, Bloom ET (2001). Effect of redox modulation on xenogeneic target cells: the combination of nitric oxide and thiol deprivation protects porcine endothelial cells from lysis by IL-2-activated human NK cells. J Immunol.

[CR123] Ulbrecht M, Couturier A, Martinozzi S (1999). Cell surface expression of HLA-E: Interaction with human beta2-microglobulin and allelic differences. Eur J Immunol.

[CR124] van’t Veer C, Golden NJ, Kalafatis M (1997). Inhibitory mechanism of the protein C pathway on tissue factor-induced thrombin generation. Synergistic effect in combination with tissue factor pathway inhibitor. J Biol Chem.

[CR125] Wallgren AC, Karlsson-Parra A, Korsgren O (1995). The main infiltrating cell in xenograft rejection is a CD4+ macrophage and not a T lymphocyte. Transplantation.

[CR126] Waterworth PD, Dunning J, Tolan M (1998). Life-supporting pig-to-baboon heart xenotransplantation. J Heart Lung Transplant.

[CR127] Weiss EH, Lilienfeld BG, Müller S (2009). HLA-E/human beta2-microglobulin transgenic pigs: protection against xenogeneic human anti-pig natural killer cell cytotoxicity. Transplantation.

[CR128] Wheeler DG, Joseph ME, Mahamud SD (2012). Transgenic swine: expression of human CD39 protects against myocardial injury. J Mol Cell Cardiol.

[CR129] Wilson CA, Wong S, Van Brocklin M (2000). Extended analysis of the in vitro tropism of porcine endogenous retrovirus. J Virol.

[CR130] Yamada K, Yazawa K, Shimizu A (2005). Marked prolongation of porcine renal xenograft survival in baboons through the use of alpha1, 3-galactosyltransferase gene-knockout donors and the cotransplantation of vascularized thymic tissue. Nat Med.

[CR131] Yan JL, Yu LY, Guo LH (2003). Human alpha galactosidase and alpha 1,2 fucosyltransferase concordantly inhibit xenoreactivity of NIH 3T3 cells with human serum. Acta Pharmacol Sin.

[CR132] Yi S, Hawthorne WJ, Lehnert AM (2003). T cell-activated macrophages are capable of both recognition and rejection of pancreatic islet xenografts. J Immunol.

[CR133] Zeyland J, Gawrońska B, Juzwa W (2013). Transgenic pigs designed to express human α-galactosidase to avoid humoral xenograft rejection. J Appl Genet.

[CR134] Zeyland J, Woźniak A, Gawrońska B (2014). Double transgenic pigs with combined expression of human α1,2**-***fucosyltransferase* and α**-***galactosidase* designed to avoid hyperacute xenograft rejection. Arch Immunol Ther Exp.

[CR135] Zhou CY, McInnes E, Copeman L (2005). Transgenic pigs expressing human CD59, in combination with human membrane cofactor protein and human decay-accelerating factor. Xenotransplantation.

